# Predictive and discriminant validity of different psychopathology and temperament scales for major psychiatric disorders – 23-year follow-up of the Northern Finland Birth Cohort 1966

**DOI:** 10.1192/j.eurpsy.2022.218

**Published:** 2022-09-01

**Authors:** J. Miettunen, A. Ahola, E. Jääskeläinen

**Affiliations:** University of Oulu, Center For Life Course Health Research, Oulu, Finland

**Keywords:** bipolar disorders, schizophrénia, Depression, temperament

## Abstract

**Introduction:**

Several psychological and psychiatric instruments have been developed to recognize or predict different psychiatric disorders.

**Objectives:**

We studied the predictive, and discriminant validity of different psychopathology scales and temperament traits for subsequent psychiatric diagnoses due to schizophrenia, bipolar and depressive disorders in a 23-year follow-up.

**Methods:**

Temperament traits, perceptual aberration, physical and social anhedonia, depression and anxiety subscales of Symptom Checklist (SCL-D and SCL-A), Hypomanic Personality Scale (HPS), Schizoidia Scale, and Bipolar II Scale were completed as part of the 31-year follow-up survey of the prospective Northern Finland 1966 Birth Cohort (n = 5006). New onset psychiatric diagnoses were followed until age of 54 years using different nationwide registers.

**Results:**

In the follow-up 28 (0.6%) individuals had diagnosis of schizophrenia, 40 (0.8%) bipolar and 405 (8.1%) depressive disorders. Several of the included scales associated statistically significantly with subsequent diagnoses. High SCL-A and SCL-D scores were strong predictors (Cohen’s d’s between 0.76 and 1.08) for schizophrenia and depressive disorders, whereas high HPS score was best predictor (d=0.67) for bipolar disorders. When comparing patient groups, schizophrenia group had low scores in reward dependence when compared with both bipolar (d=-0.80) and depressive (d=-0.66) disorders. Harm avoidance was the best trait to discriminate depressive and bipolar disorders, with higher scores in depressive disorders (d=0.48).

**Conclusions:**

Interestingly we found that differed psychopathology scales were strong but non-specific predictors for these psychiatric disorders, whereas temperament traits were useful predictors regarding discriminating these disorders. The presented scales can be used in population samples when predicting psychiatric illnesses.

**Disclosure:**

No significant relationships.

